# Does Time to Theatre Affect the Ability to Achieve Fracture Reduction in Tibial Plateau Fractures?

**DOI:** 10.3390/jcm11010138

**Published:** 2021-12-27

**Authors:** David Stuart Kitchen, Jack Richards, Peter J. Smitham, Gerald J. Atkins, Lucian B. Solomon

**Affiliations:** 1 Discipline of Orthopaedics and Trauma, Centre for Orthopaedic and Trauma Research, University of Adelaide, Adelaide, SA 5005, Australia; david.kitchen@sa.gov.au (D.S.K.); Peter.Smitham@sa.gov.au (P.J.S.); gerald.atkins@adelaide.edu.au (G.J.A.); 2 Orthopaedic and Trauma Service, Royal Adelaide Hospital, Adelaide, SA 5000, Australia; jack.richards@sa.gov.au

**Keywords:** tibial plateau fracture, fracture reduction, patient-reported outcome, fracture fixation, articular step

## Abstract

Surgical management of displaced tibial plateau fracture (TPF) is often delayed due to accompanying soft tissue injuries sustained at the time of injury. The primary aim of this study was to assess the effect of time to surgery on fracture reduction in cases of TPF. The secondary aim was to assess the effect of preoperative demographics and residual articular step on Lysholm Scores and Knee Injury and Osteoarthritis Outcome Scores (KOOS) following fixation. Patients between 2006 and 2017, managed by a single surgeon, were prospectively enrolled in the study. Reduction of articular step, defined as <2 mm, was assessed by a single blinded examiner. A total of 117 patients were enrolled, 52 with Schatzker II, 4 with Schatzker IV, and 61 with Schatzker VI fractures. Patients were followed up to a mean of 3.9 years. Analysis showed that the ability to achieve fracture reduction was negatively influenced by time to theatre, with the odds of achieving reduction decreasing 17% with each subsequent day post injury (*p* = 0.002). Furthermore, an increased time to theatre was associated with a reduced Lysholm score at one year (*p* = 0.01). The ability to achieve fracture reduction did not influence PROMs within the study period. We conclude that delay in surgical fixation negatively affects fracture reduction in TPF and may delay recovery. However, residual articular step does not necessarily influence PROMs over the mid-term.

## 1. Introduction

Patients with displaced tibial plateau fractures (TPFs) routinely undergo open reduction internal fixation with the aim to restore articular surface and joint congruency. There are limited data regarding the influence time to surgery has on fracture reduction in tibial plateau fractures. However, in acetabular fractures, which also have an association with high energy trauma and potential delays to fixation, increased time from injury to surgical intervention has been shown to affect the ability to reduce the fracture [1,2]. This time interval might be similarly important for the reduction of other articular fractures, including TPF. The decreased ability to achieve fracture reduction with increased time from injury is likely multifactorial, with the initiation of the inflammatory phase of fracture healing likely involved. Callus formation, in conjunction with local soft tissue swelling, poses challenges to achieving reduction. In addition to increased difficulty in manipulating fracture fragments as healing progresses, cancellous bone loss, occurring as early as five days post injury in the fractured tibial plateau [3], may also contribute to impaired fracture reduction. Maintenance of fracture reduction is multifactorial and may be affected by factors such as fracture severity, bone quality, surgical technique, and patient activities [4,5,6]. Indeed, such patient factors may, in some circumstances, lead to options such as total knee replacement or nonoperative management being considered [7]. Despite this, adequate reduction of articular step-off has been suggested to be the single biggest determinant of clinical outcomes in lower limb articular fractures [8,9]. Inadequate fracture reduction alters the force distributed onto the tibial plateau, leading to an axial overload and a subsequent increased rate of joint degeneration [10,11,12].

There are multiple surgical approaches that may be utilised in the fixation of tibial plateau fractures. These include anterolateral, posteromedial, direct lateral, direct medial, fibula osteotomy, and angiosome-sparing approaches [13]. Often, the choice of which approach or approaches to be used is dependent on patient factors, including the soft tissue envelope, fracture factors, and surgeon preferences [14]. Due to impaction of cancellous bone following TPFs, the use of bone graft or bone graft substitute is often utilised to fill any residual void after restoration of the articular surface [15].

TPFs are associated with soft tissue injury [16], which is a common cause for delayed surgical intervention [17] in an effort to decrease potential surgical site infections (SSIs). Indeed, compromise of the soft tissue envelope from open injury and the requirement to perform a fasciotomy in the setting of compartment syndrome are independent risk factors for surgical site infections in TPFs [18]. Whether increased time to theatre detrimentally affects the ability to reduce TPFs remains unknown. Therefore, the primary aim of this study was to assess the effect of time to surgery on the ability to reduce the articular step in TPFs. The secondary aim was to assess the effect of residual articular steps and preoperative demographics on PROMs, specifically the Lysholm and the Knee Injury and Osteoarthritis Outcome Scores (KOOS) scores, following TPF. Whether time to theatre influenced SSI in cases of TPF was also analysed.

## 2. Materials and Methods

### 2.1. Patients

This study was approved by the human research ethics committee of our institution, a tertiary referral public hospital in 2005 with updated approval in 2008 (No. 080107/2008). A consecutive cohort of patients with unilateral TPF prospectively consented to participate in the study between September 2006 and October 2017. All patients were managed under the care of a single surgeon (L.B.S.). Patients with unilateral TPFs and an articular step-off who were treated surgically were included in the study. Patients with open injuries [5], no articular depression or fracture displacement [2], and those who declined participation in the study were excluded. Follow-up review was scheduled at 6 weeks, 3 months, 6 months, 1, 2, 4, 5, 7, and 10 years, with radiographs performed at each review. All patients had physiotherapy assessment and treatment as routine with early range of motion and weight bearing encouraged. Demographic data captured included patient age, gender, preoperative smoking status, and diabetic status. Patients with postoperative surgical site infections were included for radiographic parameters but excluded from analysis of their PROMs due to known influence on patient clinical course [10]. Whilst awaiting surgery, all patients were admitted to an inpatient ward with knee immobilisation. One patient was immobilised with external fixation awaiting definitive surgery, with the remainder immobilised in either backslabs or knee immobiliser splints. During surgical fixation, a nonirradiated femoral head cancellous allograft was used in cases with residual bone loss after restoration of the articular surface. At our institution, a preference is made for earlier surgical intervention; however, a number of factors may cause delays. As a tertiary referral centre for a wide geographic area, there can be a significant delay, sometimes of several days, in patient transfer from regional centres to our own. Additional reasons for delay in surgical fixation included theatre and surgeon availability and soft tissue swelling not being amenable to surgical fixation immediately post injury.

Fracture type, time from injury, and mechanism of injury were recorded. Mechanism of injury was classified as either low energy, such as from a simple fall, or high energy, such as that sustained in a road traffic accident [10]. Fractures were classified according to Schatzker [11].

Subgroup analysis was performed on patients whose time to theatre was up to 5 days or greater than 5 days, based on previous work showing significant loss of bone in the tibial plateau in the latter group [3].

### 2.2. Radiographic Assessment

Non-weightbearing anteroposterior (AP) and lateral radiographs performed within 48 h of surgery and from each follow-up clinical review were assessed by an independent orthopaedic surgeon (D.S.K.) blinded to patient demographic factors and clinical outcomes. Assessment of the degree of reduction of articular step and preoperative joint depression was performed utilising the picture archiving and communication (PACS) software at our institution. Fracture reduction was assessed on radiographs taken on the 1st or 2nd postoperative day. Fractures were defined as reduced if residual articular steps were less than 2 mm, with steps greater than 2 mm being defined as not reduced [2,19,20]. All patients had preoperative CT scans for surgical planning and to further characterise the fracture. Overall alignment and condylar width were not included in the assessment of fracture reduction.

**Figure 1:**
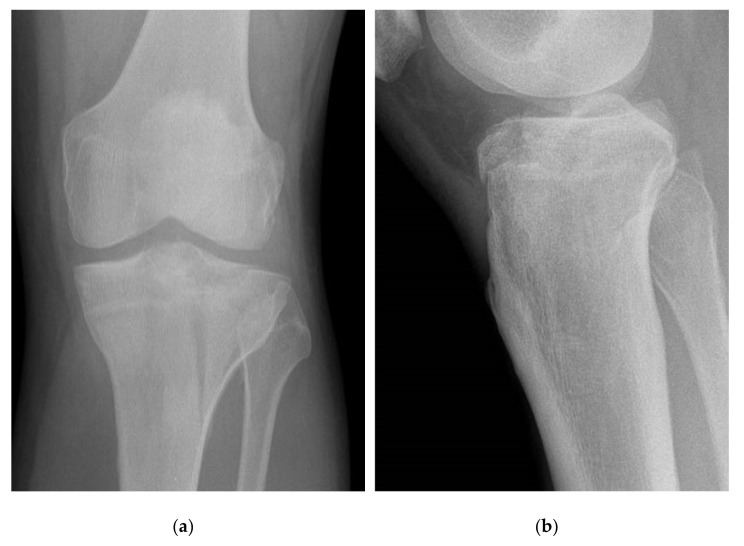

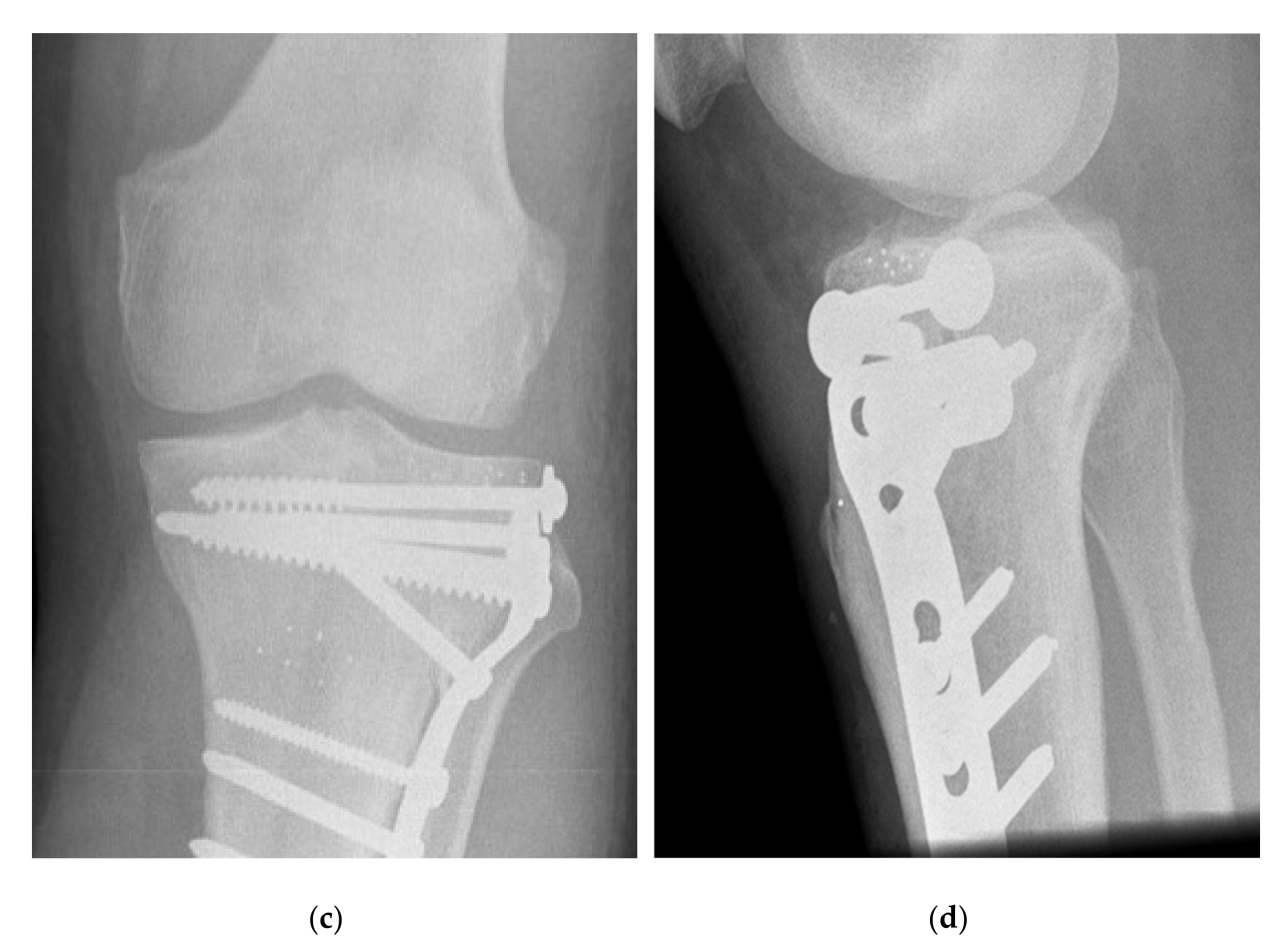
Preoperative AP (**a**) and lateral (**b**) with postoperative Day 2 AP (**c**) and lateral (**d**) radiographs of a reduced tibial plateau fracture (Schatzker II) in a patient without medical comorbidities.

**Figure 2:**
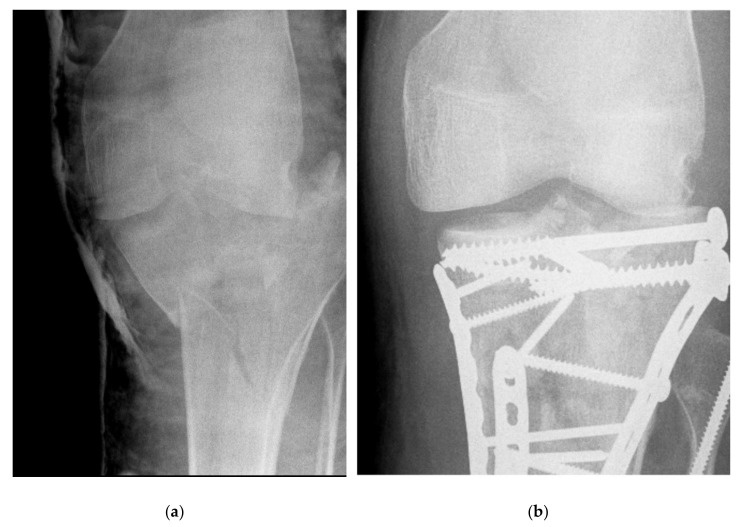

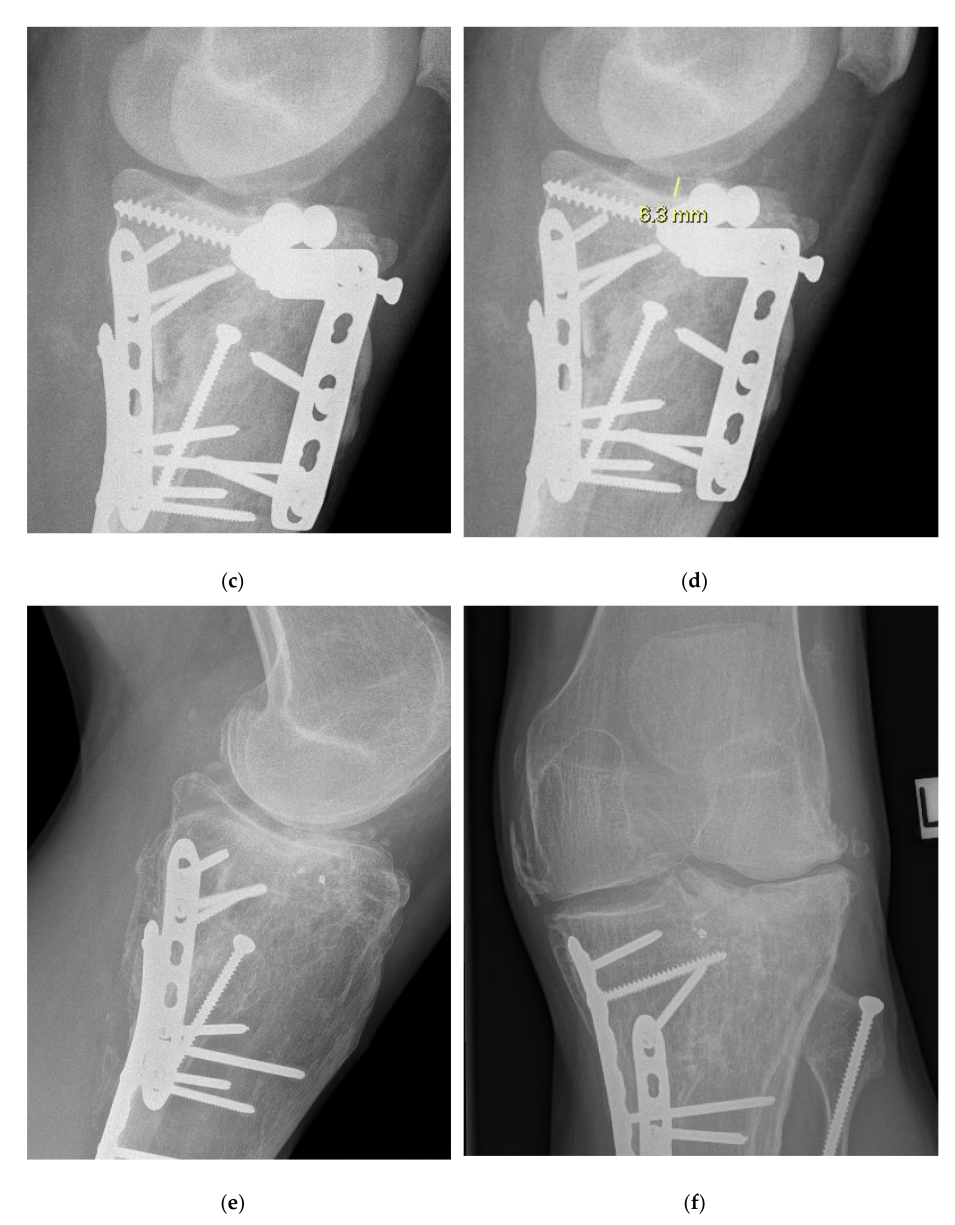
Pre- (**a**) and postoperative Day 2 (**b**) AP radiographs of a fracture that was not reduced (Schatzker VI) in a patient with Marfan syndrome. Note that the articular step is better visible on the lateral views (**c**,**d**). (**e**,**f**) Lateral and AP radiographs of the same fracture 5 years after injury following removal of the anterolateral plate and screws (removal of metalwork 21 months post primary surgery). Note the residual articular step and secondary degenerative changes. Despite fracture malreduction, the 5-year Lysholm score, KOOS pain score, and KOOS quality of life score were 80, 94.4, and 68.6, respectively.

The degree of preoperative and postoperative joint depression was assessed using both AP and lateral radiographs. The most displaced fragment was determined and analysed (Figure 1 and Figure 2).

### 2.3. Patient-Reported Outcome Measures

Questionnaires assessing the KOOS and Lysholm scores were collected at each outpatient follow-up visit. The KOOS quality of life (KOOS QOL), pain score (KOOS Pain), and Lysholm scores were analysed to determine a patient’s postinjury level of function and symptomatology. Data at 4–5 years follow up were clustered into a ‘mid-term’ time point. If patients attended at both four and five years, then the five-year time point was utilised.

### 2.4. Data Analysis

To determine the association between time to theatre and fracture reduction, both unadjusted and adjusted logistic regression models were fitted. Patient demographics, fracture type, and mechanism of injury were incorporated into the adjusted model. The effect of these factors, as well as fracture reduction and preoperative step on PROMs, was also analysed. This was undertaken utilising a two-tailed unpaired Student’s *t*-test, mixed-effect linear regression analyses, and Fisher’s Exact test, with significance assumed to be for *p* ≤ 0.05. Analyses were performed using GraphPad Prism Software (version 7; GraphPad, La Jolla, CA, USA) and Stata (version 15.1; StataCorp, College Station, TX, USA). Mean values with ranges were measured with odds ratio and 95% confidence intervals measured for the logistic regression models. The normality of the data was assessed using a Shapiro–Wilk test, with Spearman rank measured for correlation analysis.

## 3. Results

One hundred fifty-two eligible patients were identified, with 35 patients excluded, as described above. The final cohort of 117 patients consisted of 74 males and 43 females. Demographic data, including fracture type, fracture reduction, and time to theatre, are shown in Table 1. The overall mean time to theatre for all patients was 5.9 (0–26) days. Sixty-one patients were taken to theatre ≥ 5 days after their injury (52.1%), with the remaining fifty-six being operated <5 days from injury (47.9%). Demographic status, including fracture type (*p* = 0.17), mechanism of injury (*p* = 0.91), and gender (*p* = 0.48), were found not to influence time to theatre.

Patients were followed up for a mean time of 3.9 years (0.5–10). There was a loss to follow up at each time point, with 112 of 117 (96%) attending at 6 months, 108 of 117 eligible (92%) at 1 year, and 68 of 109 eligible (62%) at 4 years after surgery. These losses to follow up were due to patients failing to attend further outpatient appointments. For patients lost to follow up, on review of the reduction of their fractures, 80% of those who failed to attend at 6 months had reduced fractures, 89% for those at 1 year, and 73% at the mid-term. The percentage completion rate for PROMs scores at various time points was 70% at 6 months, 81% at 1 year, and 93% at the mid-term time point, with all patients having follow-up radiographs taken at each review.

Two patients had superficial SSI postoperatively (1.7%), and of these, one was taken to theatre Day 3 and the other Day 10 post injury.

### 3.1. Determinants of Fracture Reduction

Radiographic assessment revealed that fracture reduction was achieved in 77 cases, with 40/117 fractures being malreduced, defined as continuing to have a residual articular step-off of greater than 2 mm after surgical fixation on the immediate postoperative radiographs. However, this was deemed to be the most optimal reduction obtainable at the time of their surgery. The relevant demographics of the reduced and nonreduced cohorts are shown in Table 2. Demographic data, including preoperative joint depression (*p* = 0.47) and mechanism of injury (*p* = 0.17), were found not to influence fracture reduction (Table 3). Time to theatre was shown to have a significant relationship with the ability to achieve fracture reduction (*p* = 0.002, OR = 0.83, 95% CI = 0.74 to 0.93), with the odds of obtaining reduction decreasing 17% with every subsequent day post injury (Table 3). Patients with a time to theatre of ≥5 days were significantly less likely to have their fracture reduced when compared with those with a time to theatre of <5 days (*p* = 0.009, OR = 0.28, 95% CI = 0.11 to 0.73). Patients with reduced fractures were operated on between Days 0 and 24, with a mean time to theatre of 4.8 days following injury. Patients whose fractures were not reduced had been treated between Days 1 and 26, with a mean time to theatre of 8.0 days. Increased preoperative articular step was associated with an increased time to theatre (*p* = 0.009), but this effect was not shown when assessing patients taken to theatre <5 days with those taken ≥5 days (*p* = 0.309).

Comparable levels of joint depression were observed in both the <5- and ≥5-day time-to-theatre cohorts, with 8.08 mm (1.6–43) and 8.15 mm (1.5–54), respectively (*p* = 0.75).

### 3.2. Influence of Fracture Reduction and Type on PROMs

Neither fracture reduction nor preoperative joint depression was found to influence PROMs at any of the time points assessed (Table 4). Due to small numbers in the Schatzker IV patient cohort, the influence of fracture type and PROMs was only applied to those in the Schatzker II and VI groups. Schatzker VI fracture patients were found to have poorer Lysholm scores at the 6-month mark in comparison to Schatzker II fractures (*p* = 0.049, Table 5). There were no other significant interactions for outcome measures at any time point.

**Figure 3:**
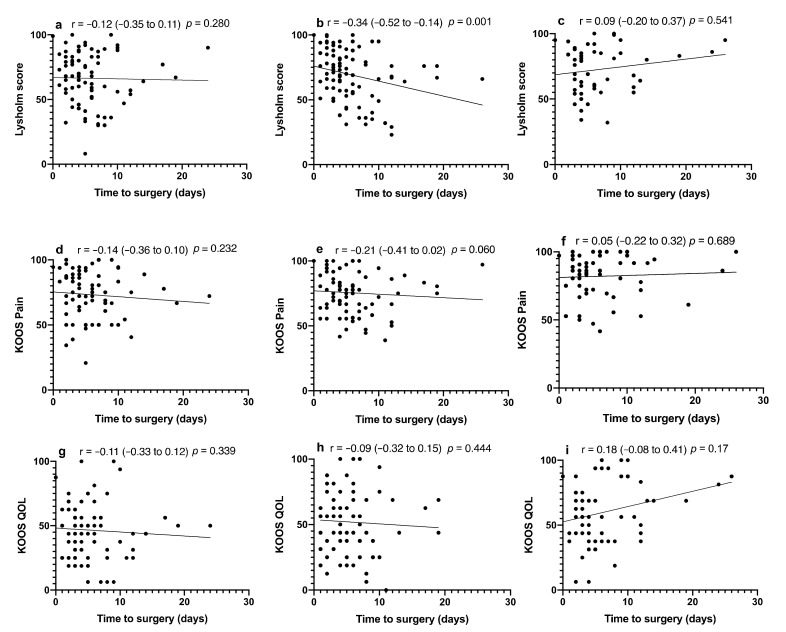
Effect of time to theatre on patient-reported outcome score. Lysholm score at (**a**) 6 months, (**b**) 12 months, and (**c**) medium term. KOOS Pain at (**d**) 6 months, (**e**) 12 months, and (**f**) medium term. KOOS QOL at (**g**) 6 months, (**h**) 12 months, and (**i**) medium term. Spearman correlation coefficients with respective confidence intervals are reported for each image.

Time to theatre showed a significant relationship with the Lysholm score at the one-year mark (*p* = 0.001) (Figure 3). Lysholm score and time to theatre at one year when grouped into <5 or ≥5 days showed that the mean score was 10.34 points lower (*p* = 0.014, 95% CI = −18.56 to −2.13) in the ≥5 days cohort. Subgroup analysis of the effect of KOOS QOL and prolonged time to theatre showed that the ≥5 days cohort had a mean score 14.23 higher than that in the <5-day group (*p* = 0.009, 95% CI = 3.63 to 24.84).

## 4. Discussion

This study demonstrated a strong negative relationship between an increased time to theatre and the ability to reduce TPFs. For each day of delay in fixation, the likelihood of achieving articular reduction decreased by 17%. Although not specifically analysed in TPF, a similar effect has been shown in acetabular fractures [2], with increased development of post-traumatic arthritic changes seen secondary to delays in surgical intervention [21]. In our study, there was a clear difference in achieving fracture reduction when assessing patients taken to theatre on or after five days from injury, when compared to those taken before Day 5, with a 72% decreased chance of reduction in those taken on or after Day 5. Many factors can be responsible for this difference, starting with the increased difficulty in manipulating fracture fragments as fracture healing progresses. We previously reported significant cancellous bone loss around the fracture site in TPFs after just 5 days of injury [3]. This very early loss of cancellous bone stock may contribute to increased difficulty in achieving fracture reduction, as fracture fragments are more susceptible to deformation during their manipulation intraoperatively. It is also possible that once bone around the fracture site has entered this catabolic state, bone loss continues post surgery, contributing to loss of reduction; however, further studies are needed to elucidate this.

The most common reasons to postpone surgical management of TPF include soft tissue swelling, surgical time, and team availability. TPFs are often associated with significant local soft tissue injury, which can compromise its blood supply, and have traditionally been thought to contribute to the increased rates of postoperative SSI in cases operated within 72 h after injury [16,22]. The effect of time to surgery on SSI in our study is inconclusive, as only two patients (1.7%), one in each group (one operated on Day 3 and the other on Day 10), developed a superficial SSI. This study suggests that if the risk of SSI is not perceived as a reason for delaying surgical management of TPFs, every effort should be made to operate early in order to provide the best chance to reduce these fractures.

The influence of articular fracture reduction on outcomes in TPFs is still a matter of debate. For example, the definition of adequate reduction of TPFs is poorly defined in the literature. The definition used in this study, that of less than a 2 mm articular step-off, one of the most commonly used in the more recent literature [19], does not take into consideration angular malalignment or condylar width. Although previous work has linked residual articular step-off with poorer PROMs when utilising a range of assessment tools, including KOOS, WOMAC, the Oxford Knee score, and the Musculoskeletal Function Assessment score [23,24], we were unable to demonstrate similar relationships between articular reduction and PROMs. There have been a number of studies that also failed to find a correlation between articular step-off and PROMs [25,26,27]. A limitation that may have influenced this lack of correlation may have been the patient completion of PROMs in our study, with between 70 and 93% of participants completing their PROMs questionnaires at each follow-up. It is worthwhile noting that there is no PROM specifically developed for TPF, which may affect the utility of the PROMs recorded in this setting. The Lysholm score was developed for knee ligament injuries [28], while the KOOS score began as a tool to measure outcome after meniscal or ACL surgery to the knee, with its use extended to post-traumatic OA and total joint arthroplasty assessment [29,30]. A trend towards improvement in PROMs at subsequent reviews was noted, which is consistent with other studies assessing outcome measures [31,32]. Based on the finding of improved one-year Lysholm scores in patients that were treated early, i.e., in those treated before Day 5 following injury, we can also speculate that earlier surgery, and therefore earlier commencement of rehabilitation, might lead to a more rapid recovery in these patients.

It is well established that patients with a more severe TPF have less favourable outcomes, with higher rates of post-traumatic arthritis and progression to total knee replacement [33]. The association between the various fracture types and PROMs is less well established, and there are suggestions that the TPF type does not correlate with PROMs, i.e., KOOS or Oxford Knee scores [23]. Our study tended to support this lack of interaction between fracture type and PROMs, though those with Schatzker II-type injuries had higher Lysholm scores at 6 months postoperative than Schatzker VI patients. This suggests that those patients with simpler fractures may report better outcomes earlier than those with more severe injuries; however, this difference may become negligible in the longer term.

This study has several limitations, including the retrospective assessment of prospectively collected data. This retrospective assessment in turn contributed to the reduced percentage completion rates for PROMs questionnaires. Participation in follow-up review remains a challenge in the trauma setting [34,35], and those with poorer outcomes may be less incentivised to attend for follow-up review, potentially skewing the data and interpretation. However, for those patients lost to follow up in this study, the vast majority of them had reduced fractures (73–87%), suggesting that fracture failure to achieve fracture reduction is not contributory to loss to follow up. Whilst SSIs are known to negatively impact patient outcomes [13], the exclusion of these patients from PROMs assessment as part of the secondary aim could be viewed as a limitation. However, given that there were only two patients (1.7%) with a surgical site infection and that the secondary aim was to assess the effect of residual articular step and patient preoperative demographics on postoperative PROMs, it was considered that a subsequent infection would potentially be a confounder.

Another study limitation may be the use of PROMs that are not specifically targeted towards the injury in question. Although KOOS and Lysholm scores are not designed to look explicitly at outcomes after TPFs, they are both commonly used in this setting [23,36]. A post hoc power analysis was not performed when assessing our secondary aim of preoperative demographics and residual articular step on PROMs, which in part can be explained due to an absence of meaningful data for a minimum clinically important difference (MCID) for tibial plateau fractures and the PROMs utilised in our study. In spite of this, the lack of a significant effect for some of the analysis could be potentially viewed as due to insufficient sample size to elucidate a statistically significant result and should be considered when interpreting this study.

Analysis of whether medial or lateral fracture fragment displacement influenced either PROMs or subsequent degenerative changes was not performed. This could be viewed as a limitation, especially given that knee stability has been suggested to be at least as important in TPF PROMs (range of motion, KOOS, WOMAC) [23,37]. A final limitation may be the heterogeneous nature of the cohort studied, with variation of fracture types and comorbidities within the group. This potential for confounders was minimised utilising multivariate analysis, to negate some of the effects of confounders. However, due to the prospective sequential recruitment of participants, it was felt that this would be a typical cohort of patients presenting to a major trauma centre with these injuries.

## 5. Conclusions

We conclude that delay in surgical fixation negatively affects the ability to reduce articular steps in TPFs. The odds of achieving fracture reduction in TPFs decreases by 17% for each day surgical intervention is delayed post injury. Interestingly, fracture type and mechanism of injury did not influence fracture reduction or time to theatre.

## Figures and Tables

**Table 1:** Patient demographics and their relationship to time to theatre.

Demographic	Subcategory	Value (Range)	Mean Time to Theatre (SD, Range) (Days)	*p* Value
Age (years) Sex		45.4 (21–78)		0.388
	Male	74	6.4 (5.0, 0–26)	0.129
	Female	43	5.1 (3.5, 1–19)	
Smoking status	Smoker	25	7.1 (5.1, 1–26)	0.114
	Nonsmoker	92	5.6 (4.3, 0–24)	
Diabetes status Schatzker type	Diabetic	5	3.4 (1.9, 2–6)	0.202
	Nondiabetic	112	6.1 (4.6, 0–26)	
	I	0		0.627
	II	52	6.0 (4.7, 0–24)	
	III	0		
	IV	4	3.8 (2.2, 1–6)	
	V	0		
	VI	61	6.0 (4.6, 1–26)	
Mechanism of injury	Low energy	39	6.0 (5.1, 2–24)	0.911
	High energy	78	5.9 (4.3, 0–26)	
Preoperative articular step (mm)		8.1 (0.5–54)		0.009
Time to theatre (days)		5.9 (0–26)		

**Table 2:** Patient demographics and their relationship to fracture reduction.

Demographic		Reduced	Unreduced	*p* Value
Age (years)		46.7 (21–78)	43.9 (24–74)	0.316
Sex	Male	44	30	0.058
	Female	33	10	
Smoking status	Smoker	13	12	0.102
	Nonsmoker	64	28	
Diabetic status	Diabetic	4	1	0.498
	Nondiabetic	73	39	
Schatzker type	II	38	14	0.077
	IV	4	0	
	VI	35	26	
Mechanism of injury	Low energy	30	9	0.074
	High energy	47	31	
Preoperative articular step (mm)		7.57 (0.5–43)	9.11 (1–54)	0.344
Mean number of plates used for fixation		1.56 (0–3)	1.84 (0–5)	0.166
Bone graft used		48	24	0.848

**Table 3:** Association between time to theatre and fracture reduction.

Exposure		Adjusted OR (95% CI)	*p* Value
Time to theatre	Days	0.83 (0.74, 0.93)	0.002
Age		1.03 (0.99, 1.06)	0.127
Sex	Male	0.64 (0.21, 1.98)	0.437
	Female	Reference	
Smoking status	Smoker	0.46 (0.16, 1.39)	0.170
	Nonsmoker	Reference	
Diabetes status	Diabetic	0.64 (0.05, 7.64)	0.722
	Nondiabetic	Reference	
Schatzker type	II	Reference	0.168
	IV	*	
	VI	0.48 (0.17, 1.36)	
Mechanism of injury	Low energy	Reference	0.173
	High energy	0.48 (0.16, 1.38)	
Preoperative articular step		0.98 (0.92, 1.04)	0.468

* perfectly predicts fracture reduction, that is, all patients with Schatzker type IV had reduced fractures.

**Table 4:** Patient-reported outcome scores in reduced and unreduced fracture patient cohorts.

	Reduced	Unreduced	*p* Value
6-month Lysholm	68.1 (30–100)	62.8 (8–92)	0.298
6-month KOOS pain	75.0 (41–100)	69.8 (21–94)	0.230
6-month KOOS QOL	48.9 (6–100)	41.2 (6–94)	0.151
1-year Lysholm	70.3 (29–100)	66.8 (23–100)	0.438
1-year KOOS pain	75.2 (39–100)	76.1 (47–100)	0.810
1-year KOOS QOL	51.7 (0–100)	52.3 (19–100)	0.918
Mid-term Lysholm	73.0 (32–100)	72.1 (34–99)	0.875
Mid-term KOOS pain	81.6 (47–100)	82.7 (42–100)	0.801
Mid-term KOOS QOL	58.0 (6–100)	62.8 (25–100)	0.436

**Table 5:** Patient-reported outcomes scores vs. fracture type (Schatzker classification).

	Schatzker II	Schatzker VI	*p* Value
6-month Lysholm	70.7 (8–100)	60.7 (9–92)	0.049
6-month KOOS pain	74.1 (21–100)	71.8 (34–97)	0.600
6-month KOOS QOL	51.1 (6–100)	41.4 (6–94)	0.067
1-year Lysholm	70 (23–100)	68.6 (29–100)	0.745
1-year KOOS pain	75.2 (42–100)	75.4 (39–100)	0.947
1-year KOOS QOL	52.3 (19–100)	50.2 (0–100)	0.708
Mid-term Lysholm	72.6 (34–100)	74.3 (32–99)	0.734
Mid-term KOOS pain	84.5 (47–100)	75.9 (56–100)	0.105
Mid-term KOOS QOL	60.2 (6–100)	51.0 (19–100)	0.314

## Data Availability

The data presented in this study are available on request from the corresponding author.
